# Bilateral Elbow Dislocation After Trauma in a Healthy Adult Female

**DOI:** 10.7759/cureus.21029

**Published:** 2022-01-08

**Authors:** Amro A Abdelrahman, Moayad A Elgassim, Ibrahim Mohamedosman A Elfaki, Khalid Y Fadul, Mohamed Abdelgadir M Elgassim

**Affiliations:** 1 Family Medicine, Hamad Medical Corporation, Doha, QAT; 2 Internal Medicine, Taylor's University Lakeside Campus, Subang Jaya, MYS; 3 Emergency Medicine, Hamad Medical Corporation, Doha, QAT

**Keywords:** traumatic bilateral elbow dislocation, simultaneous bilateral elbow dislocation, fall injury, elbow dislocation without fracture, elbow trauma

## Abstract

Bilateral elbow dislocation is a rare injury. We report a rare case of a simultaneous bilateral traumatic elbow dislocation in a 28-year-old previously healthy Kenyan female. Initial clinical assessment and plain radiographs showed the possibility of an associated fracture at the right capitulum. CT scan demonstrated bilateral fractures at the capitulum simultaneously. This case was managed conservatively through a closed reduction under procedural sedation as a joint effort of orthopedics and the emergency department. Three days later, the left above elbow backslap was removed and the patient was discharged on analgesics and referred to the outpatient clinic for regular follow-up and physiotherapy. At seven weeks, the patient reported improvement of pain bilaterally and mild stiffness at the right elbow that is continuing to improve with physiotherapy.

## Introduction

The elbow joint is the second most commonly dislocated joint in adults. The most frequent mechanism is by fall with outstretched hands with the posterolateral dislocation being the most common type. Simultaneous bilateral elbow dislocation is a rare presentation and reports of similar injuries are very limited.

We report a case of bilateral elbow dislocation with the patient's elbows being flexed during the impact of the trauma.

## Case presentation

**Figure 1:**
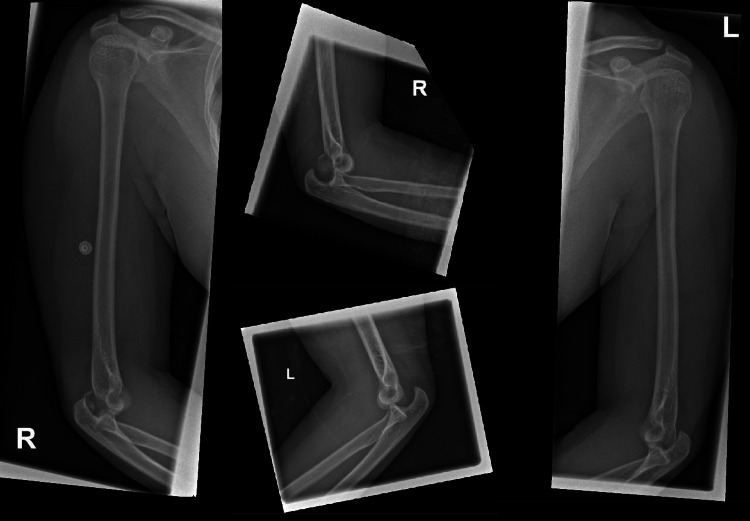
Bilateral posterolateral elbow dislocation along with non-displaced fracture involving the capitellum of the left humerus.

A 28-year-old lady of Kenyan origin, who is a maid by occupation, was brought to the emergency department with a history of falling out of a chair and landing on her hands with both her elbows in flexed positions. She had no previous history of dislocations or joint laxity, and she had no known chronic medical illness or surgical history. There was no significant family history. Clinically, the patient had pain over the joints bilaterally, along with swelling, tenderness, deformity, and a decreased range of motion. The right hand of the patient also had reduced sensation over the fifth digit prior to reduction, consistent with ulnar distribution. Plain radiograph images (Figure 1) showed bilateral posterolateral elbow dislocation along with non-displaced fracture involving the capitellum of the left humerus.

**Figure 2:**
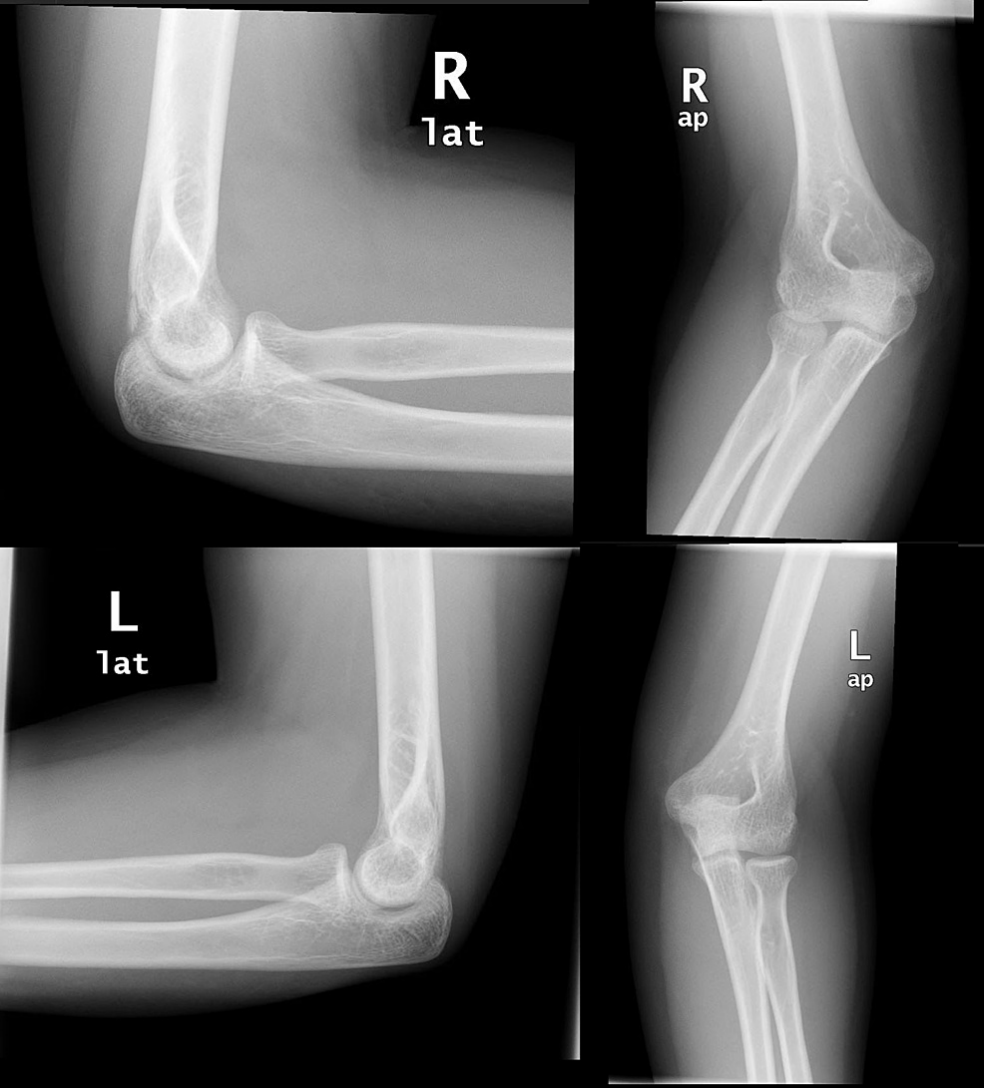
Post-reduction X-rays showing adequate reduction bilaterally.

The patient was shifted to the resuscitation area where procedural sedation was applied, and bilateral elbow reduction was achieved successfully through traction. Post-reduction X-rays showed an adequate reduction of both joints (Figure 2) and examination revealed improvement of the ulnar distribution reduction of sensation in the right hand, with no neurovascular deficits bilaterally.

**Figure 3:**
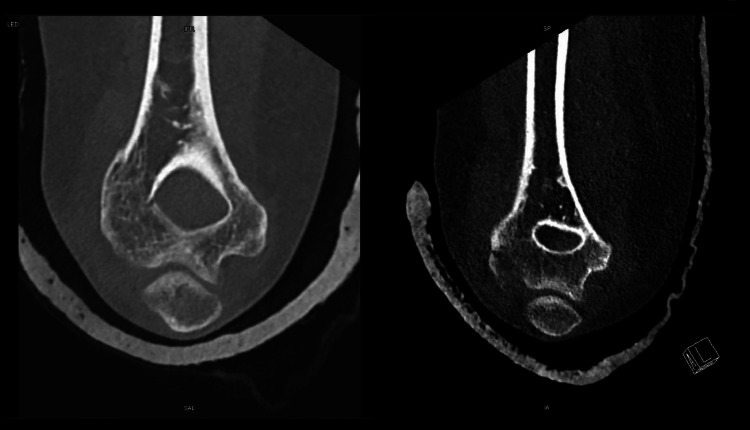
Non-displaced fractures involving the capitellums of the left and right humerus.

Bilateral backslabs were placed and the patient was admitted under orthopedics for preoperative CT imaging (Figure 3). CT imaging revealed bilateral fractures of the capitellum, with the elbow joint being correctly positioned, with no need for surgical intervention and the decision by the orthopedics team was to go for conservative treatment for elbow fracture bilaterally. Her backslabs were removed and she was referred to their outpatient clinic. Her follow-up in one week showed a full range of motion and function in both elbows with no further complaints.

## Discussion

Our patient was a 28-year-old lady with no underlying conditions who suffered from bilateral elbow dislocation after falling from a chair height and landing on her hands with her elbows in a flexed position.

Bilateral elbow dislocation is a rare presentation that occurs due to the specific circumstances in which the patient’s body weight acts upon the extended elbow joints [1] and is commonly associated with female gymnastics or cases of joint hyperlaxity [1,2], while in contrast, our patient has no similar background history, and her elbow joints were flexed during the impact.

Similar cases of bilateral dislocation are commonly associated with high-energy trauma when compared to unilateral dislocation [3]. Cases described in the literature are attributed to falling off a bike or falling off a broken ladder [3], which is not very different from our patient who fell off a chair.

The treatment for such cases of dislocations differs on a case-to-case basis, depending on the severity of the case and the presence of associated injuries. Simple unilateral dislocations of the elbow are generally treated with closed reduction of the joint and temporary immobilization for a period of one to three weeks [4,5].

A bilateral cast for the same period of time leads to severe impairment of the patient’s daily function, which may require hospitalization [4,6]. Furthermore, this immobilization of the elbow for a period over three weeks carries a risk of joint stiffness [5], which happens as a result of joint fibrosis [7], which could be catastrophic for both elbows simultaneously. The patient can benefit from early mobilization. In our patient’s one-week follow-up, she demonstrated no complications and retained a full range of motion and function of both elbows, and her backslabs were removed.

Limitations

One of the major difficulties we faced with this case is the scarcity of resources in the literature that can be referenced. Over the past 10 years, only a handful of cases were reported with a similar presentation, which limits us to referring to older references that may be outdated for the present day.

## Conclusions

Bilateral elbow dislocation is a rare injury with minimal literature citing. Careful history taking of background comorbidities as well as mechanism of injury needs to be done followed by a thorough clinical examination to rule out other associated injuries of bones and neurovascular deficits. Whenever feasible, radiographical imaging should provide multiple views of the joints as occult fractures may be difficult to find and should include the long bones proximal and distal to look for further bony injuries. Reduction of these dislocations may be done in the emergency department if there are no complicating fractures while immobilizing the joint and with timely routine follow-up within a week. A repeated full examination should be done to assess for possible complications of reduction during follow-ups with orthopedics, and prolonged immobilization of the joint should be avoided to prevent further complications.

Overall, bilateral elbow dislocation is a rare injury that requires specialized care and proper treatment. We report a 28-year-old patient with this infrequent injury. Further studies are needed regarding flexed bilateral elbow dislocation.
